# Field-free magnetization reversal by spin-Hall effect and exchange bias

**DOI:** 10.1038/ncomms10854

**Published:** 2016-03-04

**Authors:** A. van den Brink, G. Vermijs, A. Solignac, J. Koo, J. T. Kohlhepp, H. J. M. Swagten, B. Koopmans

**Affiliations:** 1 Eindhoven University of Technology, PO Box 513, Noord-Brabant, 5600 MB Eindhoven, The Netherlands; 2 SPEC, CEA, CNRS, Université Paris-Saclay, CEA Saclay 91191 Gif-sur-Yvette, France

## Abstract

As the first magnetic random access memories are finding their way onto the market, an important issue remains to be solved: the current density required to write magnetic bits becomes prohibitively high as bit dimensions are reduced. Recently, spin–orbit torques and the spin-Hall effect in particular have attracted significant interest, as they enable magnetization reversal without high current densities running through the tunnel barrier. For perpendicularly magnetized layers, however, the technological implementation of the spin-Hall effect is hampered by the necessity of an in-plane magnetic field for deterministic switching. Here we interface a thin ferromagnetic layer with an anti-ferromagnetic material. An in-plane exchange bias is created and shown to enable field-free S HE-driven magnetization reversal of a perpendicularly magnetized Pt/Co/IrMn structure. Aside from the potential technological implications, our experiment provides additional insight into the local spin structure at the ferromagnetic/anti-ferromagnetic interface.

## Abstract (web summary)

Future information storage technology may exploit electrical currents to write the states of ferromagnetic nanoelements via spin torque effects. Here, the authors demonstrate such behaviour promoted by exchange bias from an interfaced antiferromagnet, which may help overcome practical device limitations.

## Graphical abstract

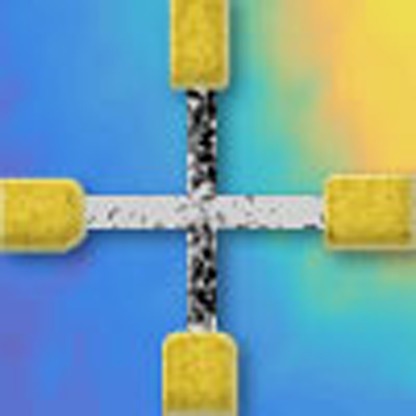

In recent years, magnetoresistive random-access memory has matured to the point where it is considered a serious contender for dynamic random-access memory (DRAM) replacement^1^,^2^,^3^. Key advances have been the discovery of the magnetic tunnel junction (MTJ) for reading^4^,^5^ and the spin-transfer torque (STT) effect for writing^6^,^7^, a significant improvement over magnetic-field-based designs^8^. However, the current density and energy dissipation involved in STT-driven magnetization reversal remains problematic^9^, even with the advent of more efficient tunnel junctions that exhibit perpendicular magnetic anisotropy (PMA)^10^.

Research efforts to improve on the STT writing paradigm explore the use of electric fields^11^, multi-ferroics^12^, perpendicular polarizers^13^ and spin–orbit torques^14^. The latter category is dominated by devices employing the spin-Hall effect (SHE)^15^,^16^,^17^, which has been shown to be a viable method of spin injection in recent experiments^18^,^19^,^20^,^21^. Magnetization reversal using only SHE was demonstrated for in-plane magnetized MTJs^20^ but remains challenging in perpendicularly magnetized MTJs, which are more relevant due to higher data storage density. Additional symmetry breaking is required to allow the in-plane polarized spin current generated from the SHE to deterministically switch out-of-plane magnetized devices. In the laboratory, this has been achieved by applying an in-plane magnetic field^18^,^19^ or creating an anisotropy gradient^22^, but these methods are not suitable for practical applications.

Here we address this issue by interfacing the perpendicularly magnetized layer with an anti-ferromagnetic material, creating an in-plane exchange bias (EB) along the current flow direction. We demonstrate SHE-driven magnetization reversal using only the intrinsic in-plane magnetic field caused by this EB. Gradual magnetization reversal is observed and attributed to the poly-crystalline nature of the anti-ferromagnet, which agrees with numerical simulations.

## Results

### Perpendicular anisotropy and in-plane EB

**Figure 1:**
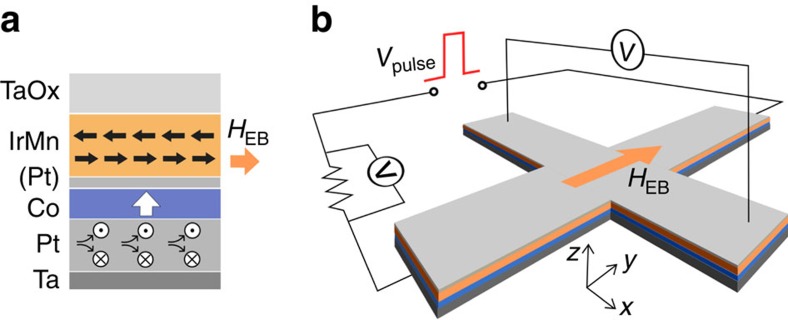
Schematic sample layout. (**a**) Cross-section of the deposited stack (labels indicate the deposited materials), showing the magnetic easy axis of the Co (white arrow), simplified spin structure of the IrMn (thick black arrows), EB field *H*_EB_ (orange arrow) and spin current generated from a charge current running through the Pt (circles). (**b**) Hall cross structure consisting of two 10 × 1 μm rectangles with a certain *H*_EB_ (orange arrow) and measurement scheme comprising a voltage pulse generator (*V*_pulse_), a series resistor and two voltmeters (*V)*.

Experiments were performed on Ta (1)/Pt (3)/Co (0.7)/Pt (0.3)/IrMn (6)/TaOx (1.5) stacks (nominal thicknesses in nanometres), patterned into Hall crosses. Samples were field cooled to set the EB along the 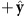 direction (see Fig. 1), as described in the Methods section. The presence of both PMA and in-plane EB was verified by carrying out magneto-optic Kerr effect (MOKE) and superconducting quantum interference device–vibrating sample magnetometry (SQUID–VSM) measurements on unstructured samples after annealing (see Supplementary Note 1 and Supplementary Figs 1 and 2). Out-of-plane MOKE measurements show square loops with a coercive field *μ*_0_*H*_C_≈40 mT and negligible EB. In-plane SQUID–VSM measurements show an EB field of *μ*_0_*H*_EB_≈50 mT. Furthermore, the saturation magnetization is measured at *M*_S_=1.2 MA m^−1^ with a saturation field *μ*_0_*H*_K_≈1.0 T, indicating a substantial PMA of *K*_eff_≈6.0 × 10^5^ Jm^−3^.

### Proof-of-principle

**Figure 2:**
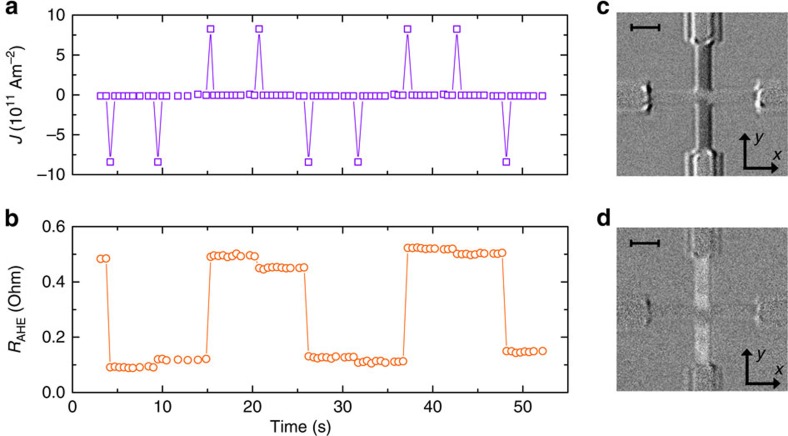
Switching by current pulses. (**a**) Applied current density pulse pattern and (**b**) resulting anomalous Hall resistance *R*_AHE_. Switching is observed for one current polarity in either state, without any applied magnetic field. Differential Kerr microscopy images of the microwire after switching to (**c**) the low *R*_AHE_ state and (**d**) the high *R*_AHE_ state confirming the magnetization reversal. Scale bars, 2.5 μm (the top left corners).

Samples are subjected to a sequence of current pulses along the 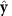 direction, in the absence of applied magnetic fields. Through the SHE, a current in the 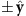 direction should generate a spin current polarized in the 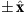 direction for positive spin-Hall angles, as in Pt^18^. Such a spin current can switch the magnetization from 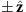 to 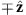, provided that both current density and the effective magnetic field along the 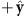 direction are large enough. Switching in the other direction should occur only if the current polarity is reversed. We successfully demonstrate this behaviour in our devices, using 50 μs current pulses (*J*=8 × 10^11^ Am^−2^) in the sequence shown in Fig. 2a. No external magnetic field is present during this measurement. Deterministic switching is clearly observed on reversing the current polarity, as seen in both anomalous Hall effect resistance (*R*_AHE_) and MOKE measurements (Fig. 2). Moreover, subsequent pulses of equal polarity have little effect on the magnetization. Furthermore, varying the pulse duration between 1 and 100 μs was found not to affect the end result significantly. It is noteworthy that samples without Pt dusting layer show similar results, exhibiting deterministic magnetization reversal without applied magnetic field (see Supplementary Note 2 and Supplementary Fig. 3). From this proof-of-principle measurement, it is evident that the EB provides sufficient effective magnetic field to facilitate deterministic SHE-driven magnetization reversal.

### Detailed study of magnetization reversal

Two more subtle features, visible in Fig. 2, were found to be reproducible and require further investigation. First, the magnetization shows a small jump in response to repeated current pulses of the same polarity, which is unexpected. Second, the MOKE images suggest that magnetization reversal in the centre of the Hall cross is less complete than outside this region. Taking into account that the current density is ∼30% lower in the centre of the Hall cross (see Supplementary Note 3 and Supplementary Fig. 4), it appears that magnetization reversal in the absence of magnetic fields is incomplete, especially at lower current densities.

**Figure 3:**
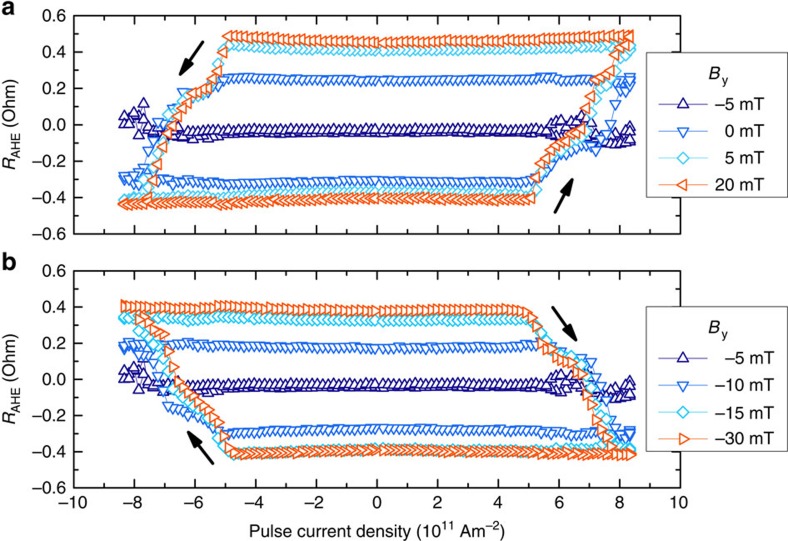
Current density sweeps. Anomalous Hall resistance *R*_AHE_ measured during pulse current density sweeps for various applied in-plane magnetic fields *B*_y_. The magnetic field enhances deterministic (**a**) upward and (**b**) downward switching. The arrows indicate the sweep direction.

To explore this effect in more detail, we sweep the pulse current density from high negative values to high positive values and back. In addition, we apply a magnetic field *B*_y_ along the 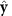 direction to investigate how this affects the magnetization reversal. The resulting *R*_AHE_(*J*_pulse_) curves (Fig. 3) show several interesting features.

The total change in magnetization after a current density sweep, Δ*R*_AHE_, is found to strongly depend on *B*_y_. For *B*_y_=−5 mT, we find that Δ*R*_AHE_ is negligible, implying a complete absence of deterministic switching. This result is expected for a spin-Hall current in the absence of an effective magnetic field, suggesting that the effective EB field is compensated by *B*_y_ at this point. It is noteworthy that this compensation point is not equal to the EB field measured in unstructured samples, as will be discussed later. Increasing *B*_y_ in either direction is seen to gradually increase Δ*R*_AHE_: partial reversal is observed in the range −15 mT to +5 mT. This behaviour is remarkably different from devices without an EB, which have been shown to switch abruptly at a certain critical field^18^,^19^.

Furthermore, a finite slope is clearly observed in the switching loops, representing a gradual change in *R*_AHE_ for increasing *J*_pulse_. This suggests that magnetization reversal is not uniform but occurs in many small domains, each with a different critical current density for deterministic switching. Again, this behaviour is radically different from samples without an EB, which show more sudden magnetization reversal (see Supplementary Note 4 and Supplementary Fig. 5). It is worth noting that substantial domain wall propagation is not observed, in agreement with electron microscopy studies in comparable magnetic/anti-ferromagnetic bilayers^23^,^24^.

Finally, the current density required for magnetization reversal is identical for up–down and down–up switching, confirming that there is no preferential direction along the 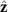 axis. The vertical offset is negligible in all loops, indicating that the entire measured region is affected by the current. For the *B*_y_=−5 mT trace, for instance, this implies that a large current density produces equal amounts of up and down magnetized domains, such that *R*_AHE_=0.

We note that substantial Joule heating occurs at higher current densities. By comparing the resistivity during current pulses to a calibration measurement, we estimate that temperatures may briefly rise as high as 650 K (see Supplementary Note 5 and Supplementary Fig. 6). However, we found no evidence for an irreversible change to the EB magnitude during our experiments, suggesting that the pulse time is too short for thermally activated processes to affect the anti-ferromagnetic ordering (see Supplementary Fig. 7).

### Systematic variation of magnetic field and current density

**Figure 4:**
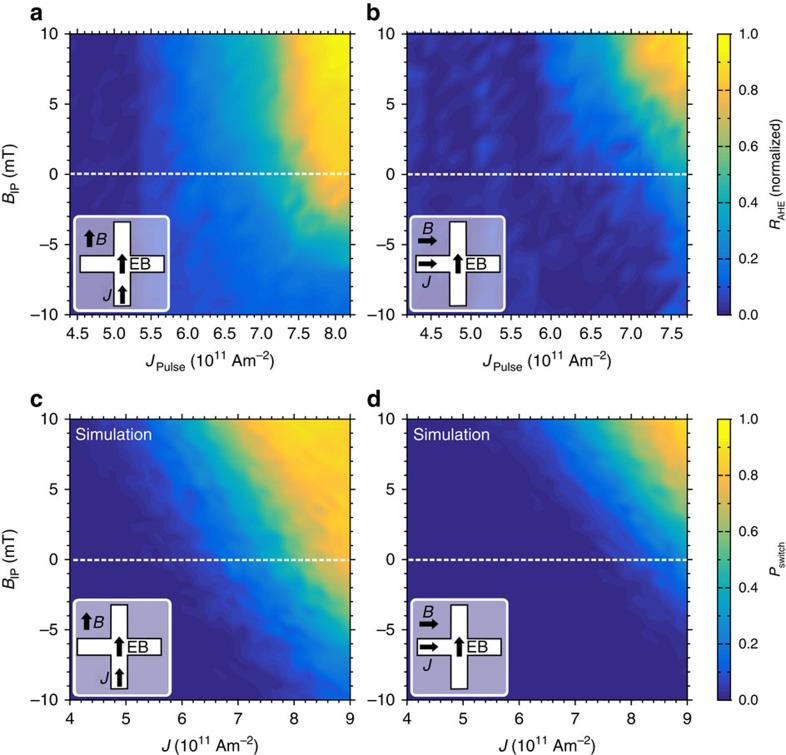
Magnetization reversal versus current density and magnetic field. Measurements show the normalized change in anomalous Hall resistance *R*_AHE_ with current density *J* and applied magnetic field *B*_IP_ (**a**) parallel and (**b**) perpendicular to the EB direction. The small difference in horizontal axes is caused by a resistance difference between the two directions of the Hall cross. Evaluation of the LLG equation, including Joule heating and local variations in the EB direction and magnitude, produces comparable phase diagrams for both the (**c**) parallel and (**d**) perpendicular configurations when computing the switch probability *P*_switch_.

To further explore magnetization reversal driven by SHE and in-plane EB, we systematically vary the pulse current density and assisting magnetic field, both parallel and perpendicular to the EB direction. For each combination of field and pulse current density, the magnetization is first saturated in the 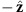 direction. The change in *R*_AHE_ before and after pulse application is measured and normalized to the largest recorded Δ*R*_AHE_, resulting in the phase diagrams shown in Fig. 4a,b. The diagrams agree with SHE-driven switching experiments^18^,^19^ and provide several key insights into the effect of the EB, as detailed below.

First, we look at the *B*_IP_=0 traces in the phase diagrams. Confirming the proof-of-principle result, near-complete magnetization reversal is observed for strong current pulses along the EB direction (Fig. 4a). Furthermore, a maximum of 50% magnetization reversal is attained when measuring perpendicular to the EB direction (Fig. 4b) even for high current densities, indicating random rather than deterministic switching.

Second, the perpendicular-to-EB measurement resembles the parallel-to-EB measurement shifted vertically by *B*_IP_≈6 mT, close to the effective EB observed in Fig. 3. However, for intermediate current densities Δ*R*_AHE_ is larger parallel to the EB, as can be seen from the light blue area in Fig. 4a. This implies that partial magnetization reversal, at intermediate current densities, is also easier along the EB direction.

Third, we find that the phase diagrams can be reproduced by numerical evaluation of the Landau–Lifshitz–Gilbert (LLG) equation (see Methods) implementing the SHE as an in-plane polarized spin current and the EB as an effective magnetic field (Fig. 4c,d). Importantly, the agreement between simulations and experiments is improved by selecting the EB magnitude and direction from appropriate distributions, as discussed below.

## Discussion

**Figure 5:**
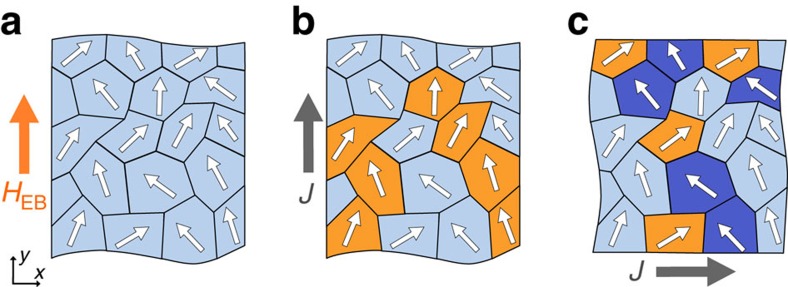
Simplified sketches of grains within the anti-ferromagnetic layer. The small arrows represent the uncompensated spin direction in each grain at the interface with the ferromagnet. (**a**) Situation after field-cooling, showing an average EB field *H*_EB_ (orange arrow). Partial magnetization reversal occurs in the adjacent ferromagnetic layer after (**b**) a current pulse along the EB direction or (**c**) a current pulse perpendicular to the EB direction. Switched regions are indicated in orange and blocked regions are indicated in dark blue. Grey arrows show the current flow direction.

Concluding our measurements, deterministic switching of perpendicular magnetization by an in-plane current was demonstrated in the absence of magnetic fields. The magnetization reversal process is not complete, however, as concluded from measurements using an additional in-plane magnetic field. Partial switching appears to be intrinsic to SHE-driven magnetization reversal under small applied magnetic field. We believe that the physical origin of this effect must be sought in the local structure of the anti-ferromagnetic layer, which produces conditions subtly different from an applied magnetic field, which is inherently homogeneous. Sputtered IrMn has a polycrystalline morphology^25^, which complicates the simplistic picture of EB painted in Fig. 1a. During annealing, anti-ferromagnetic spins align to the field-cooling direction on average, but the actual spin direction within a grain is bound to local crystallographic axes^25^ as sketched in Fig. 5a. Furthermore, variations in grain size and orientation affect the local magnitude of the EB^26^. This local spin structure, present in any EB system, appears to affect SHE-driven magnetization reversal especially.

A current pulse can induce deterministic switching via SHE only if there is sufficient effective magnetic field along the current direction. We propose that, at a given current density and small applied in-plane field, these conditions hold only for a subset of regions where the local uncompensated spin direction has sufficient component along the current direction, as illustrated in Fig. 5b. This explains why partial magnetization reversal is observed at small in-plane magnetic fields. Furthermore, grains can exist where the local EB is against the current flow direction if one measures perpendicular to the EB direction (Fig. 5c). Magnetization reversal is suppressed in such grains, which explains the reduced Δ*R*_AHE_ observed in Fig. 4b for intermediate current densities.

As mentioned before, our experiments can be reproduced by numerical evaluation of the LLG equation. We implement the local spin structure of the anti-ferromagnetic layer by averaging over many simulations while drawing the EB direction from a distribution appropriate for a cubic polycrystalline material. This produces a range of applied fields and current densities where partial magnetization reversal occurs, significantly improving the agreement with experiments over simulations with a uniform EB of 5 mT. The agreement is further improved by drawing the local EB magnitude from a *χ*_3_-distribution to account for grain size variations and by implementing Joule heating to match resistance data (see Supplementary Note 6 and Supplementary Figs 9–11). Further experimental research may elucidate the role of anti-ferromagnetic grains in current-driven experiments, but is deemed beyond the scope of this work. SHE-driven magnetization reversal, aside from its technological relevance, may thus provide a unique tool in understanding the local spin structure at ferromagnetic/anti-ferromagnetic interfaces.

Finally, the apparent distribution in EB magnitude and direction partially explains the discrepancy between the EB field of 50 mT observed in SQUID–VSM measurements and the 5 mT effective in-plane field observed in current-driven switching experiments. Furthermore, brief Joule heating may reversibly reduce the EB magnitude (see Supplementary Note 5 and Supplementary Fig. 8), but no reports on this subject exist in the literature. In addition, it is known that patterned structures can exhibit reduced EB^27^ and the used lift-off process may reduce the film quality. Improving fabrication conditions to obtain a more uniform (ideally single-crystalline) anti-ferromagnetic layer should resolve this issue, allowing for reliable field-free binary switching using the SHE. Future memory devices may even employ the SHE of anti-ferromagnetic metals^28^,^29^ to enable efficient readout of the magnetic state using an MTJ structure.

In summary, we have demonstrated field-free SHE-driven magnetization reversal by interfacing an out-of-plane magnetized Co layer with a Pt spin-Hall injection layer and an IrMn exchange-biasing layer. A proof-of-principle measurement shows field-free switching and exhibits all expected symmetries. The amount of magnetization reversal is found to increase when applying an additional in-plane magnetic field. This observation can be attributed to the polycrystalline nature of the anti-ferromagnet, as confirmed by simulations. Improving the crystalline structure of the anti-ferromagnetic layer could lead to reliable binary switching. We believe that these measurements provide a significant breakthrough in applied spintronics, as well as a unique probe for the local spin structure of polycrystalline anti-ferromagnetic materials.

## Methods

### Sample preparation

Samples were fabricated on polished, thermally oxidized silicon substrates using DC sputtering at a base pressure around 10^−8^ mbar. The deposited stack (Fig. 1a) consists of Ta (1)/Pt (3)/Co (0.7)/Pt (0.3)/Ir_20_Mn_80_ (6)/TaOx (1.5), with nominal thicknesses in nanometres. The Pt dusting layer was inserted to enhance the PMA and was found not to be detrimental to the EB, in agreement with the literature^30^. Layer thicknesses were chosen after careful optimization, as detailed in Supplementary Note 7 and Supplementary Figs 12 and 13. A Pt thickness of 3 nm, in particular, optimizes the SHE efficiency (see Supplementary Note 8 and Supplementary Fig. 14). Using a lift-off electron-beam lithography procedure, the stack is patterned into Hall crosses (Fig. 1b) consisting of two overlapping 10 × 1 μm rectangles. A small pad at each extremity of the Hall cross connects to thick Ti/Au electrodes (not shown in the figure), to allow for electrical contact. The completed structures are then placed in a 2.0 T in-plane magnetic field along one of the Hall bar axes, annealed at 225 °C for 30 min and finally field cooled to set the EB direction. For the device discussed in the main text, the resistance along the EB direction of the Hall cross was measured to be 899 Ω at room temperature.

### Magnetic characterization

Thin-film magnetic characteristics were studied using two methods: polar MOKE and VSM implementing a SQUID. The MOKE is measured on a custom-built laser setup, allowing for high-precision measurement of Kerr rotation using a photo-elastic modulator and lock-in amplifier. The used SQUID–VSM is a Quantum Design MPMS 3.

### Experimental setup

The magnetization reversal process was studied using an Evico Kerr microscope in polar mode, allowing for high-resolution digital imaging of the out-of-plane magnetization component. In addition, an Agilent 33250A pulse generator was used to apply voltage pulses and a small DC voltage to allow for *R*_AHE_ measurements, providing an accurate measure of the average out-of-plane magnetization in the junction area. The pulse current could be determined by monitoring the voltage drop over a resistor in series with the device. Current densities are computed by dividing the current over the total metallic cross-sectional area of the microwire, which is 11 nm × 1 μm.

### Current shunting estimation

Current shunting in the Hall bar structure is estimated using the COMSOL Multiphysics 5.1 finite element solver, as discussed in Supplementary Note 3.

### Numerical simulations

Following the approach of our earlier work^17^, magnetization dynamics are simulated by evaluating the LLG equation:


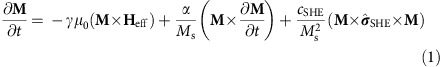


where **M** is the free layer magnetization, *γ* is the electron gyromagnetic ratio, *μ*_0_ is the vacuum permeability, **H**_eff_ is the effective magnetic field, *α* is the Gilbert damping coefficient and 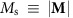 is the saturation magnetization. The spin-Hall torque coefficient is given by 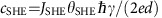, where *J*_SHE_ is the current density running underneath the free layer, *θ*_SHE_ is the spin-Hall angle of the material underneath the free layer, *ħ* is the reduced Planck constant, *e* is the elementary charge and *d* is the free magnetic layer thickness. The effective magnetic field **H**_eff_ comprises six contributions as follows: (i) the applied magnetic field; (ii) the EB field; (iii) the magnetic anisotropy field; (iv) the demagnetization field, which is approximated as that of an infinite thin film; (v) a Langevin thermal field, to account for thermal fluctuations; and (vi) an Oersted field generated by *J*_SHE_, which is approximated by that of an infinite surface current. Current shunting effects are neglected. Joule heating can be included by assuming that heat proportional to 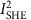 is absorbed, while Newtonian cooling to the environment (at 300 K) takes place. Appropriate coefficients are used to produce temperature profiles matching experiments, that is, an equilibrium temperature of 650 K for *J*_SHE_=8 × 10^11^ Am^−2^, which is reached within a few nanoseconds. Further simulation details, regarding the numerical implementation and the values of used parameters, are included in Supplementary Note 9. The EB direction and magnitude distributions are shown in Supplementary Figs 15 and 16, respectively.

## Author contibutions

A.v.d.B. designed the experiment, carried out exploratory measurements, performed simulations and wrote the manuscript. G.V. carried out the main experiment and processed the data. A.S. and J.K. assisted in measurements and interpretation of the data. J.T.K., H.S. and B.K. supervised the project. All authors revised the manuscript.

## Additional information

**How to cite this article:** van den Brink, A. *et al*. Field-free magnetization reversal by spin-Hall effect and exchange bias. *Nat. Commun.* 7:10854 doi: 10.1038/ncomms10854 (2016).

## Supplementary Material

Supplementary Information Supplementary Figures 1-16, Supplementary Notes 1-9 and Supplementary References
