# Supplementary material for: Field-free magnetization reversal by spin-Hall effect and exchange bias
Source: Nat Commun. 2016 Mar 4;7:10854. doi: 10.1038/ncomms10854 (PMC5411711; doi:10.1038/ncomms10854)
Supplement: Supplementary Information — Supplementary Figures 1-16, Supplementary Notes 1-9 and Supplementary References [file ncomms10854-s1.pdf]

Supplementary Note 1.   Thin film magnetization measurements

Thin film magnetic characteristics were studied using polar Magneto-Optic Kerr Effect (MOKE) and SQUID Vibrating Sample Magnetometry (SQUID-VSM) measurements, both before and after the field-cooling process. Here, we show the data obtained for the Ta (1) / Pt (3) / Co (0.7) / Pt (0.3) / IrMn(6) / TaOx(1.5) stack (nominal thicknesses in nm) discussed in the main text.

After deposition, MOKE measurements show a typical double-loop behavior (Supplementary Figure 1a). A random pattern of up- and downward out-of-plane (OOP) magnetization arises in the Co layer during deposition, and is transferred to the IrMn layer. This creates regions of positive and negative exchange bias (EB) as seen in the MOKE loop. A uniform OOP EB can be obtained by heating the sample to 225 °C while applying an OOP magnetic field larger than the coercive field. This is shown in Supplementary Figure 1b; a MOKE measurement taken after OOP field cooling at 0.2 T.

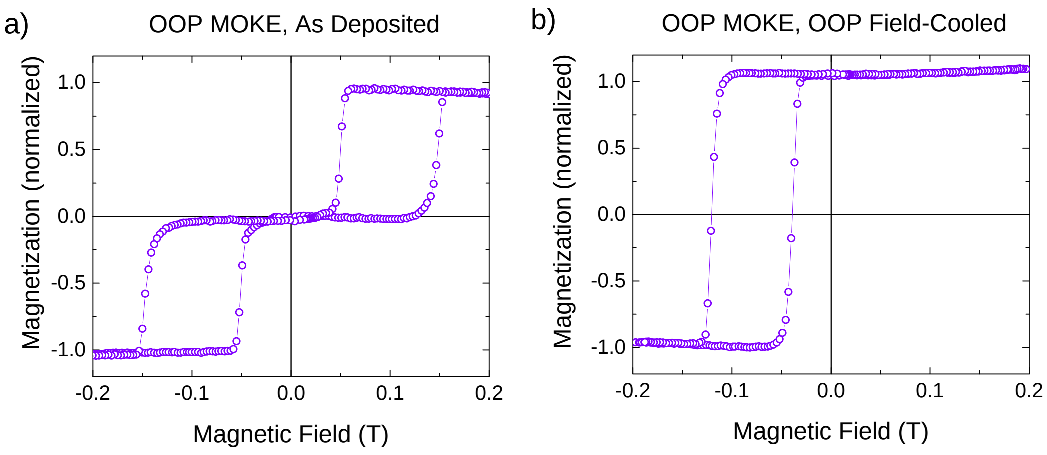

Supplementary Figure 1 | Thin film magnetization versus out-of-plane field. Out-of-plane component of the magnetization as a function of applied out-of-plane magnetic field, measured by polar MOKE in the **(a)** as-deposited and **(b)** out-of-plane field-cooled states.

Creating an in-plane EB in samples with perpendicular magnetic anisotropy (PMA) is more difficult, as the intrinsic anisotropy field needs to be overcome to force the magnetization of the Co layer in the in-plane direction during field-cooling. We therefore applied a large in-plane field of 2.0 T while heating the sample to 225 °C and field-cooling over a period of 30 minutes. Afterwards, OOP MOKE measurements (Supplementary Figure 2a) show full remanence, a substantial coercivity $\mu_0 H_\mathrm{c} \approx 40$ mT, and negligible EB in the out-of-plane direction. The squareness of the loop is clear evidence for a substantial perpendicular magnetic anisotropy. In-plane SQUID-VSM measurements (Supplementary Figure 2b) show an in-plane EB of $\mu_0 H_\mathrm{EB} \approx$ 50 mT and a saturation magnetization $M_s = 1.2 \times 10^6 \mathrm{Am}^{-1}$ with $\mu_0 H_\mathrm{K} \approx 1$ T, indicating an effective PMA of $K_\mathrm{eff} \approx 6.0 \times 10^5$ Jm^{-3}. A slight opening is visible in the SQUID-VSM cycle, probably caused by a slight misalignment between the sample surface and the measurement direction.

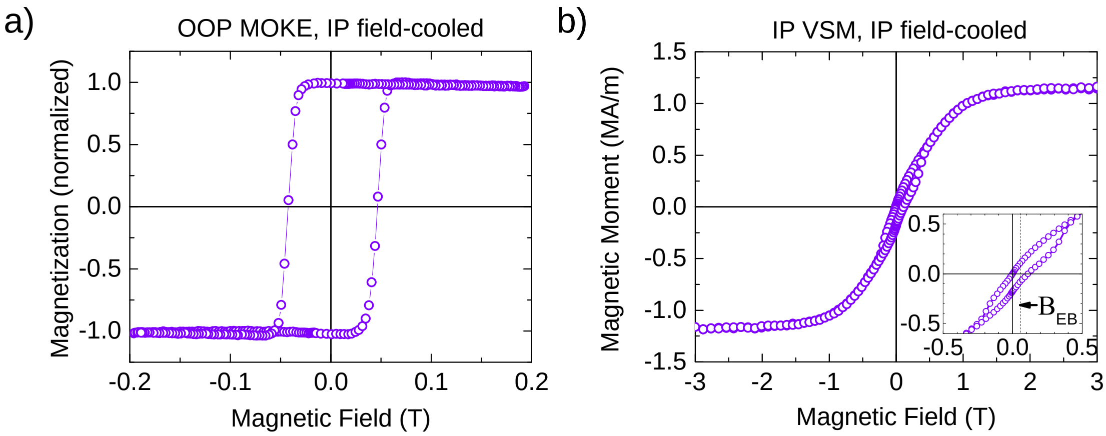

Supplementary Figure 2 | Hysteresis loops after in-plane field cooling. Thin film magnetization versus magnetic field measured by **(a)** polar MOKE along the out-of-plane axis and **(b)** SQUID-VSM along the in-plane direction. The sample shows fully remanent out-of-plane magnetization with a coercive field of 40 mT as well as an exchange bias field of 50 mT in the in-plane direction (see inset).

Supplementary Note 2. Proof-of-principle without dusting layer

The main text focuses on measurements performed in a Pt/Co/Pt/IrMn sample, where a Pt dusting layer was added between the Co and IrMn to improve the anisotropy. Similar measurements were performed on samples without dusting layers, with similar results. The proof-of-principle experiment performed on a Ta(0.5) / Pt (4) / Co (1.25) / IrMn (6) / TaOx (1.5) (nominal thicknesses in nm) is shown in Supplementary Figure 3, and also exhibits field-free magnetization reversal driven by an in-plane current. Note that the resistance of this device is substantially lower (532 Ω along the EB direction of the Hall cross) owing to the increased Pt and Co thicknesses. These devices were not as robust against high current densities, so construction of a complete phase diagram proved impossible. The main text therefore focuses on a sample with a Pt dusting layer, which proved to be more stable at high current densities.

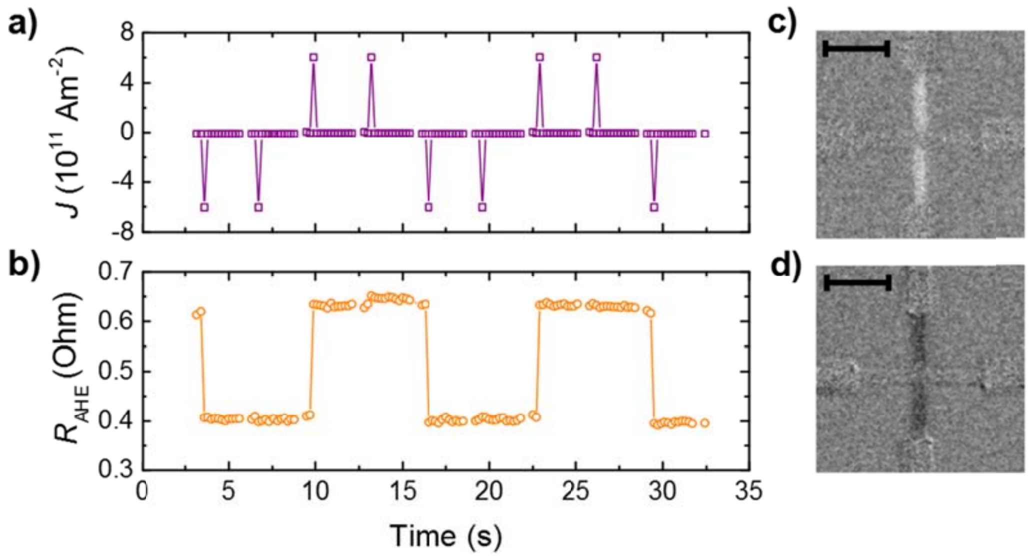

Supplementary Figure 3 | Proof-of-principle measurement without dusting layer. Current pulses are applied to a Ta (0.5) / Pt (4) / Co (1.25) / IrMn (6) / TaOx (1.5) sample, demonstrating that **(a)** a train of current pulses produces **(b)** magnetization switching as expected from the SHE. Kerr microscopy images show the magnetization after switching to **(c)** the up-state and **(d)** the down-state. The scale bars in the top left corners span 5 μm.

Supplementary Note 3.   Current shunting effect

As discussed in the main text, several experiments suggest that magnetization reversal in the center of the Hall cross occurs at a higher applied current than reversal in the rest of the microwire. To explain this behavior, we examine the current density distribution in the Hall cross using COMSOL simulations. Due to the current shunting through the inactive arms of the Hall cross, the current density in the center is reduced by roughly 30%, as shown in Supplementary Figure 4. Referring to the main text, such a significant reduction is indeed expected to affect magnetization reversal in our experiments.

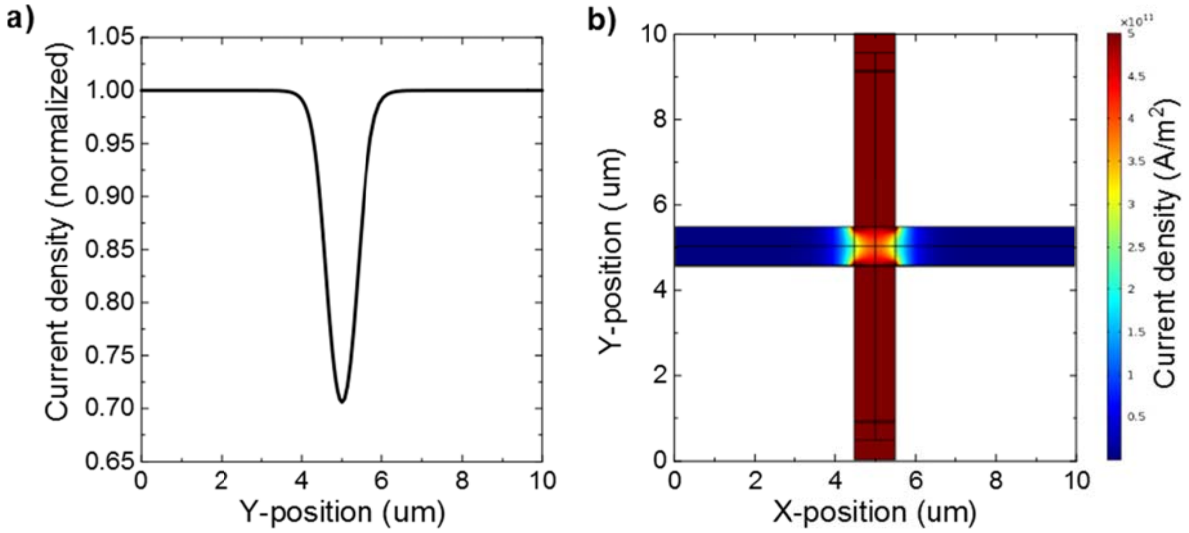

Supplementary Figure 4 | Current shunting simulations. (a) Normalized current density along the central axis in the y-direction. The current density in the center is about 30% lower. **(b)** 2D current density plot of the Hall cross, where current is flowing along the y-direction.

Supplementary Note 4. Magnetic reversal without exchange bias

As discussed in the main text, gradual magnetization reversal is observed when sweeping the current density from high negative to high positive values and back, even in the presence of additional in-plane magnetic fields. We proposed that the local spin structure of the IrMn causes a distribution of effective local magnetic fields. To test this hypothesis, we created a Hall cross sample without an anti-ferromagnetic layer, composed of Ta (4) / Pt (3) / Co (1.2) / Ta (5), with nominal thicknesses in nm. In this sample switching is found to be abrupt (Supplementary Figure 5), suggesting rapid domain wall propagation across the measured region. This is markedly different from the exchange-biased samples, where no evidence of coherent domain wall propagation was found. Even if slow domain wall propagation occurs, the steps must be far below the submicron range accessible in the Kerr microscope, in agreement with existing studies on similar bilayers[1,2]. We conclude that the gradual magnetization reversal is not related to domain wall motion (or device geometry in general) and results directly from the IrMn layer.

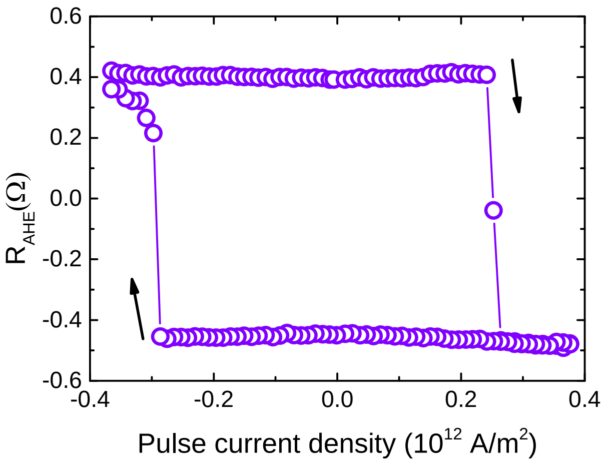

Supplementary Figure 5 | Hysteresis loop in a sample without exchange bias. Anomalous hall resistance R_{AHE} of a Ta(4)/Pt(3)/Co(1.2)/Ta(5) microwire, recorded while sweeping the applied pulse current density from high negative values to high positive values and back under application of a -10 mT magnetic field along the current flow direction. Sudden magnetization reversal is observed at a critical current density below $0.3\times10^{12}\mathrm{Am}^{-2}$

Supplementary Note 5. Joule heating and temperature

Given the high current densities, Joule heating and temperature-related effects could be of great importance in our experiments. We measured the resistance of a microwire (identical in dimensions and composition to the sample discussed in the main text) while ramping the temperature using a uniformly heated Argon flow (Supplementary Figure 6a). Comparing this calibration data to resistance measurements performed during the pulsed current experiments, we were able to quantify the amount of Joule heating (Supplementary Figure 6b) and found that the temperature may rise as high as 650 K for the highest used current densities.

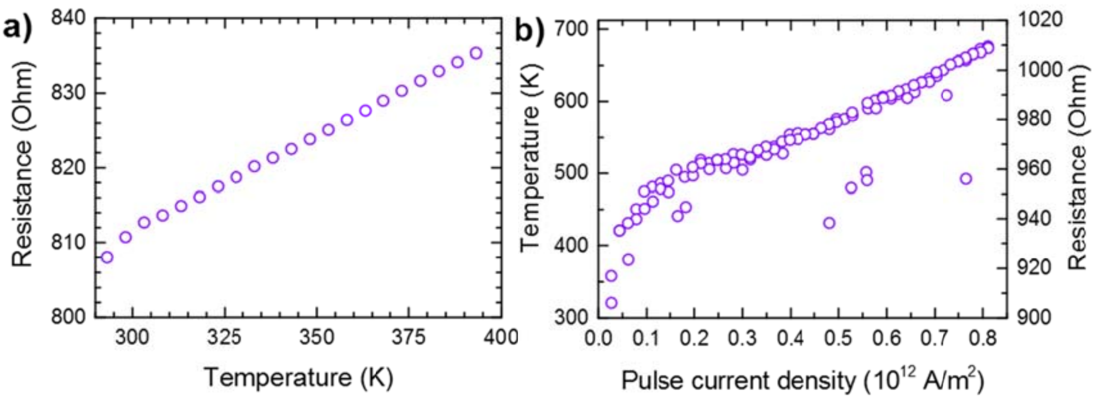

Supplementary Figure 6 | Temperature dependent resistance data. (a) Resistance versus temperature measured by controlled heating of a nanowire similar to the one used in the main experiments and **(b)** temperature computed from resistance as a function of pulse current density for the sample discussed in the main text.

Due to Joule heating, the temperature may thus briefly exceed the blocking temperature of thin IrMn films (450 K) and even approach the Néel temperature (690 K)[3]. If such a temperature were to be sustained for extended periods of time, the exchange bias of our samples would vanish and reset upon cooling. This is not observed: our measurements show clear evidence of exchange bias at high current densities and the exchange bias is still (equally) present after

recording the full phase diagrams. Crucially, although elevated temperature influences the magnetization reversal process, the used current pulses (typically 50 μs) are too short to irreversibly affect the exchange bias. Anti-ferromagnetic re-ordering is a thermally activated process that scales logarithmically in time. The relevant time-scale τ is given by[4]:

$$\frac{1}{\tau} = \nu_0 \exp\left[-\frac{E_\mathrm{b}}{k_\mathrm{B}T}\right] \tag{1}$$

with the attempt frequency $\nu_0 = 10^9$ s^{-1}, E_b the energy barrier for anti-ferromagnetic grain reversal, k_B Boltzmann's constant and T the temperature. We know that our samples are stable for at least several weeks at room temperature, so that $\tau \sim 10^6$ s at $T_0 = 300$ K, yielding $E_\mathrm{b} \approx 34.5 \times k_\mathrm{B}T_0$. At a temperature of 650 K, we find $\tau \sim 10$ ms (see Supplementary Figure 7), which is three orders of magnitude larger than the current pulse duration.

Still, we cannot exclude that briefly approaching the Néel temperature has an instantaneous, reversible effect on the exchange bias. One could speculate, for instance, that this would reduce the effective exchange bias due to a reduction of the anti-ferromagnetic ordering within grains, as illustrated in Supplementary Figure 8. This would contribute towards our findings that the effective exchange bias measured in our switching experiments (roughly 5 mT) is lower than the 50 mT value we measured in thin films at room temperature. However, to our knowledge, such an effect has never been reported on before.

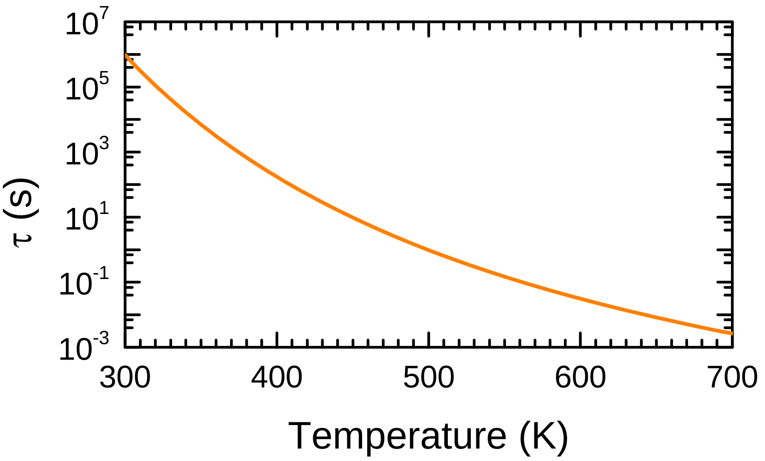

Supplementary Figure 7 | Exchange bias relaxation time-scale. Estimated exchange bias relaxation time-scale τ as a function of temperature, based on a thermal stability of several weeks at room temperature.

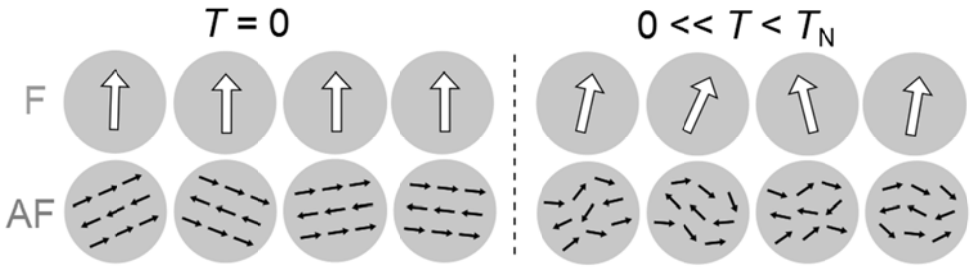

Supplementary Figure 8 | Reversible reduction of exchange bias near the Neél temperature. Sketch of ferromagnetic (F) and anti-ferromagnetic (AF) grains (grey circles) comprised of strongly coupled F spins (white arrows) and AF spins (black arrows). Increasing the temperature T from $T = 0$ K towards the Neél temperature T_N hypothetically leads to a reversible reduction of anti-ferromagnetic ordering within the AF grains.

Supplementary Note 6. Simulation results

Evaluation of the LLG equation, as described in Supplementary Note 9, typically yields magnetization trajectories as shown in Supplementary Figure 9. A large number of simulations can be combined to create phase diagrams, such as those shown in Supplementary Figure 10 for 0 K. The left-hand (right-hand) panels show the situation where the current flow is along (perpendicular to) the mean EB direction. Compared to a uniform 5 mT exchange bias, both direction and magnitude variations improve the agreement with experimental data (c.f. Figure 4, main text). Adding ambient temperature and Joule heating further improves the agreement with experimental data (Supplementary Figure 11), whereas temperature-related effects alone (assuming uniform EB) cannot fully explain the observations.

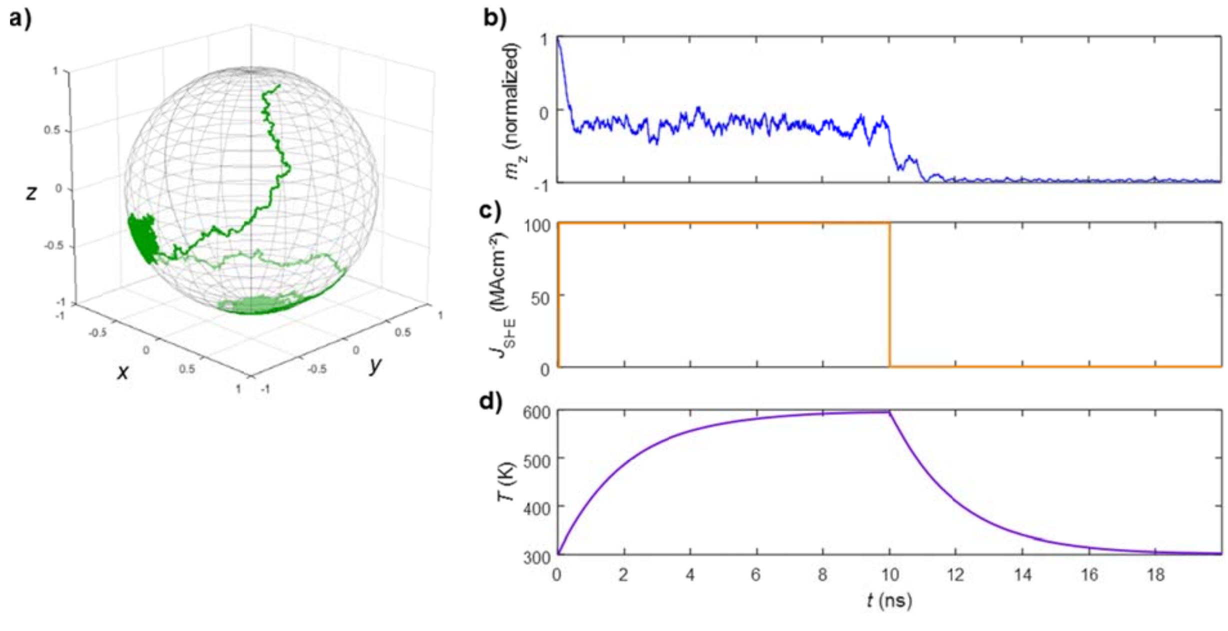

Supplementary Figure 9 | Typical macrospin simulation result with Joule heating enabled. Separate plots show **(a)** the 3D trajectory of the (normalized) magnetization, as well as the corresponding traces of **(b)** m_z, **(c)** J_{SHE}, and **(d)** temperature.

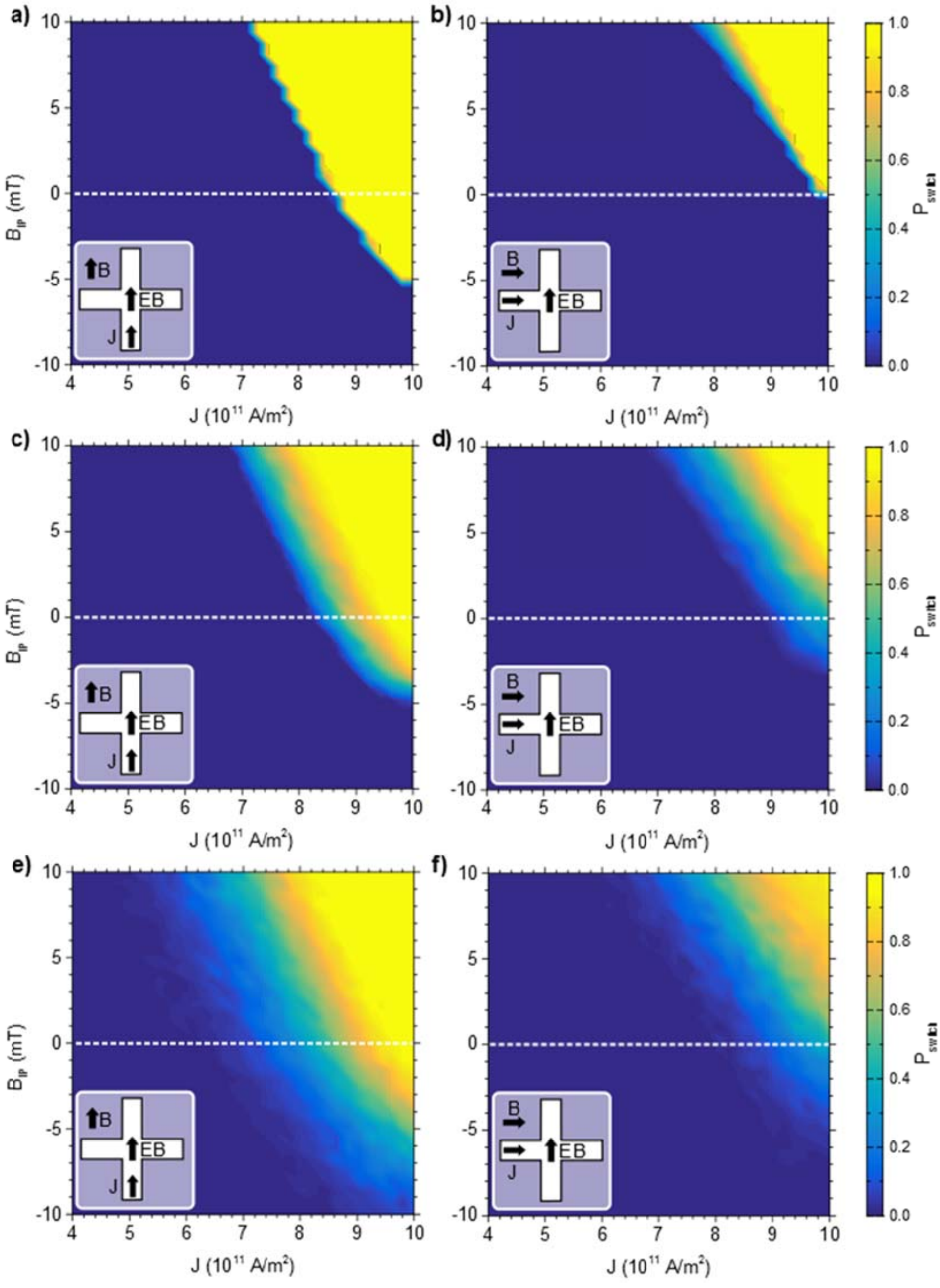

Supplementary Figure 10 | Macrospin simulation results at zero temperature. The switching probability P_{switch} is plotted as a function of current density J and magnetic field B_{ip} along the current direction, for currents running parallel **(a,c,e)** and perpendicular **(b,d,f)** to the exchange bias direction. Specific parameters for each set of panels are: **(a,b)** a 5 mT uniform exchange bias, **(c,d)** a 5 mT EB with angular spread, and **(e,f)** a χ_3-distributed exchange bias with angular spread. For each (J,B_{ip}) point, P_{switch} is computed by averaging over 256 simulations.

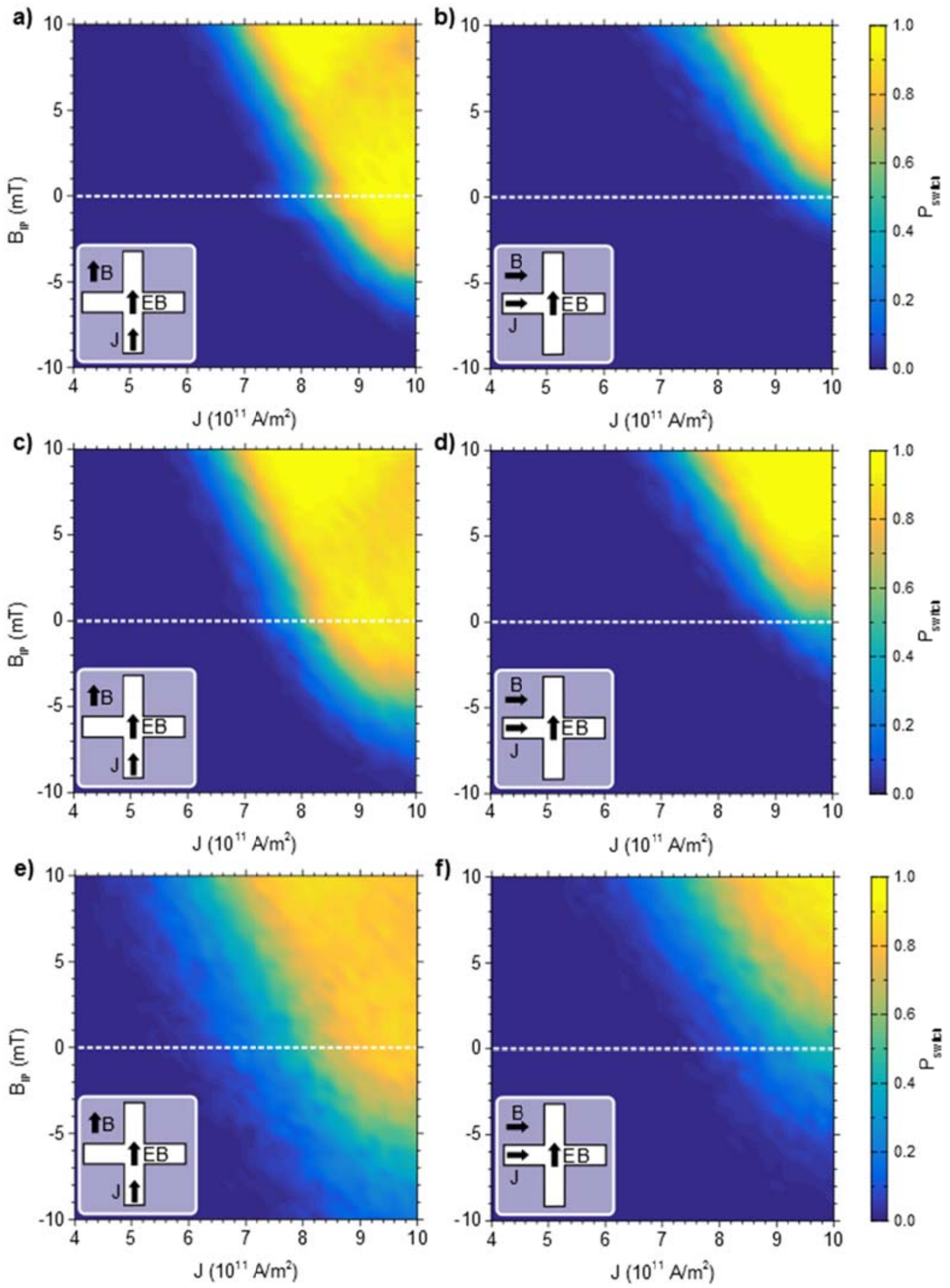

Supplementary Figure 11 | Macrospin simulations results at nonzero temperature. The switching probability P_{switch} is plotted as a function of current density J and magnetic field B_{ip} along the current direction, for currents running parallel **(a,c,e)** and perpendicular **(b,d,f)** to the exchange bias direction. Specific parameters for each set of panels are: **(a,b)** a 5 mT uniform exchange bias and fixed temperature of $T = 300$ K, **(c,d)** a 5 mT uniform exchange bias, $T_{env} = 300$ K, and Joule heating, and **(e,f)** a χ_3-distributed exchange bias with angular spread, $T_{env} = 300$ K, and Joule heating. For each (J,B_{ip}) point P_{switch} is computed by averaging over 256 simulations.

Supplementary Note 7. Stack optimization

The composition of the material stack used in our experiments is the result of careful optimization of all layer thicknesses. Relevant results are presented here, to provide an overview of the effect of each layer thickness.

Deposition starts with a Ta seed layer, which is commonly used to improve film quality[5] and was found to significantly increase the PMA in our samples. The thickness of this buffer layer was minimized to reduce current shunting effects. This reduces the PMA, but we found that a 1 nm Ta seed layer suffices for our measurements.

The Pt thickness of 3 nm maximizes the spin-Hall effect (SHE); see Supplementary Note 8.

The Co layer was chosen as thin as possible, to maximize both the PMA and the susceptibility to spin currents injected from the interface. MOKE measurements were performed on a sample with a variable Co thickness, which was subjected to the in-plane field cooling process. As shown in Supplementary Figure 12, a thickness of 0.7 nm yields the largest coercivity and full remanence ($M(0)/M_s$), indicating that a substantial PMA is obtained for this Co thickness.

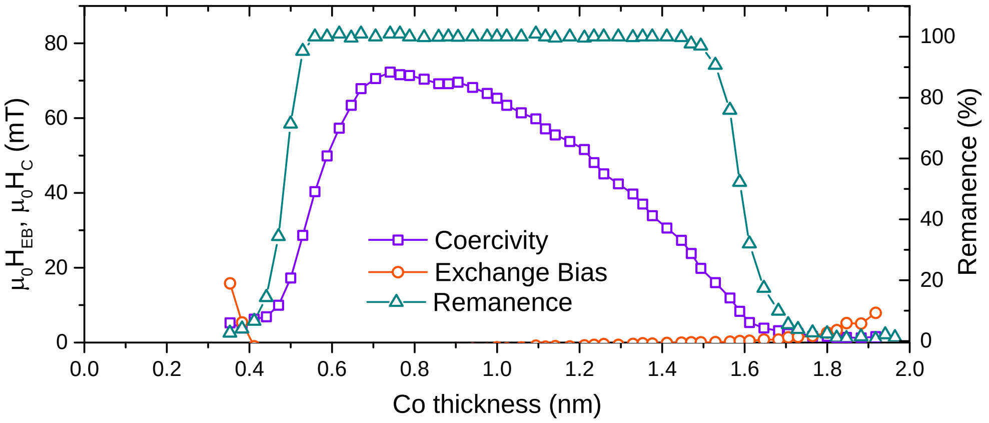

Supplementary Figure 12 | Magnetic characteristics as a function of Co thickness. The exchange bias H_{EB}, coercive field H_c, and remanence are shown as a function of cobalt thickness in a Ta (3) / Pt (3) / Co (0-2) / Pt (0.3) / IrMn (6) / Pt (3) wedge sample, measured using polar MOKE in the out-of-plane direction after annealing in the in-plane direction at 225°C in a 2T magnetic field for 30 minutes.

The thickness of the IrMn layer is crucial to obtain a large and stable EB. We created a sample with a variable IrMn thickness, and annealed it at 225°C in a 0.2 T out-of-plane magnetic field for 30 minutes. This allows us to measure the EB and coercivity as a function of IrMn thickness using polar MOKE, which we found to be a good measure for the properties of an in-plane annealed sample. As shown in Supplementary Figure 13, the highest EB is obtained for an IrMn thickness of 6 nm. Note that the coercivity peak and negligible EB indicate that the EB is unstable for reduced thicknesses. The reduction in EB observed at higher thicknesses can probably be attributed to changes in microstructure or domain structure in the IrMn[6].

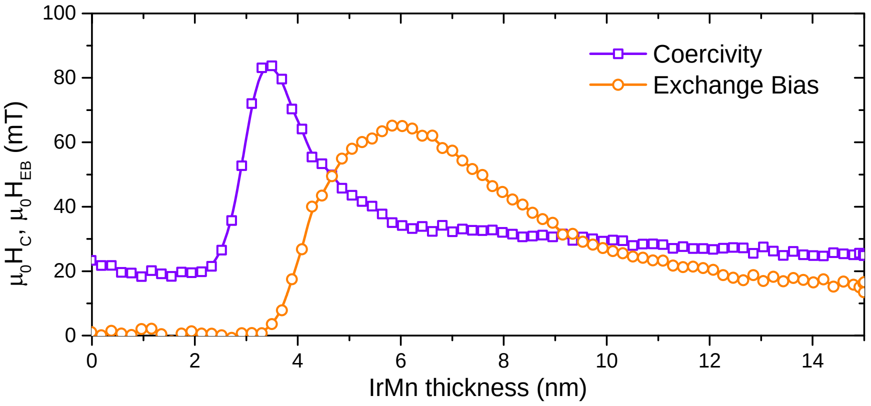

Supplementary Figure 13 | Coercivity and exchange bias versus IrMn thickness. Graphs of the exchange bias H_{EB} and coercive field H_c as a function of IrMn thickness in a Ta (3) / Pt (4) / Co (1.5) / IrMn (0-15) / Pt (2) wedge sample, measured along the out-of-plane direction after annealing in the out-of-plane direction at 225°C in a 0.2 T magnetic field for 30 minutes. The sample shows close to 100% remanence across the entire IrMn thickness range.

A 0.3 nm Pt dusting layer was inserted between the Co and IrMn layers to increase the PMA of the Co layer. Interestingly, this dusting layer was found to significantly reduce the chance of device breakdown at high current densities. Note that this thin layer is not expected to contribute significantly to the net spin current due to scattering, as discussed in Supplementary Note 8.

Finally, the stack is capped with a 1.5 nm Ta layer which is allowed to oxidize naturally, producing a protective yet transparent and non-conductive capping layer.

Supplementary Note 8. Spin current considerations

Owing to the spin-Hall effect, a vertical spin current density J_s can be generated from a planar charge current J_e in materials with a nonzero bulk spin-Hall angle $\theta_{SH} \equiv J_s/J_e$. For our thin Pt films, we use the reported[7] value of θ_{SH}= 0.07. Note that extensive debate exists on this subject, which is beyond the scope of this publication. For ultrathin films, the thickness of the metallic layer affects the net spin current. Spin accumulations are created at the interfaces with adjacent layers, causing spin diffusion that reduces the net spin current significantly if the film thickness is of the order of the spin-diffusion length λ_{sf}. Following the approach of Liu *et al.*[7], we take λ_{sf} = 1.4 nm for Pt and model the net spin current as

$$J_s = J_e \theta_{SH} (1 - \operatorname{sech} \frac{d}{\lambda_{sf}}), \qquad (2)$$

where *d* is the Pt layer thickness. From this perspective, a thicker Pt layer is beneficial as it improves the net spin current. However, this also increases the total electric current I_e required to produce a certain current density J_e, which increases Joule heating and thus the risk of device breakdown. To solve this trade-off, we compute the spin current J_s as a function of Pt thickness *d* while constraining J_e to maintain a constant total current I_e. The result of this computation is shown in Supplementary Figure 14. Current shunting through other metallic layers in the stack is taken into account, using a basic calculation where the stack is regarded as a parallel resistor network with appropriate resistances based on bulk conductivities. The optimum value for the Pt thickness is thus determined to be between 3 and 4 nm.

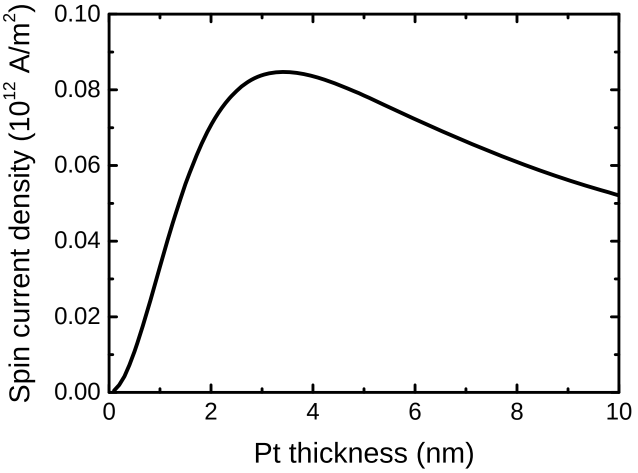

Supplementary Figure 14 | Spin current density as a function of Pt thickness. The spin current density is computed for constant total current I_e. Spin diffusion and current shunting effects are taken into account using simple models. The optimum Pt thickness is found to be between 3 and 4 nm.

In our calculations, we only take into account the spin current generated from the thick Pt layer. This is justified, as contributions from the other layers are negligible. By the diffusion mechanism here, the contribution from the 0.3 nm Pt dusting layer is about thirty times smaller than the contribution from the 3 nm Pt layer. The Ta seed layer is also very thin, and its local conductivity will be significantly reduced due to elastic scattering at the substrate interface. Finally, although IrMn may exhibit a spin-Hall angle comparable to Pt[8], its conductivity is more than an order of magnitude lower. Therefore, its contribution to the total current density and total spin current is negligible compared to the Pt.

Supplementary Note 9. Simulation details

As discussed in the main text, following the approach of our earlier work[9], magnetization dynamics are simulated by solving the Landau–Lifshitz–Gilbert (LLG) equation[10]:

$$\frac{\partial \mathbf{M}}{\partial t} = -\gamma \mu_0 (\mathbf{M} \times \mathbf{H}_{\text{eff}}) + \frac{\alpha}{M_s}\left(\mathbf{M} \times \frac{\partial \mathbf{M}}{\partial t}\right) + \frac{c_{\text{SHE}}}{M_s^2}(\mathbf{M} \times \hat{\boldsymbol{\sigma}}_{\text{SHE}} \times \mathbf{M}) \tag{3}$$

with **M** the free layer magnetization, γ the electron gyromagnetic ratio, μ_0 the vacuum permeability, $\mathbf{H}_{\text{eff}}$ the effective magnetic field, α the Gilbert damping coefficient, and $M_s \equiv |\mathbf{M}|$ the saturation magnetization. The spin-Hall torque coefficient is given by $c_{\text{SHE}} = J_{\text{SHE}}\theta_{\text{SHE}}\hbar\gamma/(2ed)$, J_{SHE} the spin-Hall effect current density running underneath the free layer, θ_{SHE} the spin-Hall angle, $\hbar$ the reduced Planck constant, e the elementary charge, and d the free magnetic layer thickness. The Oersted field generated by J_{SHE} is approximated by that of an infinite surface current, whereas current shunting effects are neglected. Joule heating can be included by assuming that heat proportional to I_{SHE}^2 is absorbed while Newtonian cooling to the environment (at 300 K) takes place. Appropriate coefficients are used to produce temperature profiles matching experiments, i.e. an equilibrium temperature of 650 K for $J_{\text{SHE}} = 8 \times 10^{11}$ Am^{-2}, which is reached within a few nanoseconds.

The effective magnetic field $\mathbf{H}_{\text{eff}}$ comprises six contributions: the applied magnetic field $\mathbf{H}_{\text{appl}}$, the exchange bias field $\mathbf{H}_{\text{EB}}$, the effective anisotropy field $\mathbf{H}_{\text{ani}} = 2K_{\text{U}}/(\mu_0 M_{\text{s}})\hat{\mathbf{z}}$, with K_{U} the uniaxial anisotropy energy density, the demagnetizing field $\mathbf{H}_{\text{D}}$ which is approximated for an infinite thin film, an Oersted field $\mathbf{H}_{\text{Oe}}$ generated by the current running through the spin-Hall injector, and a Langevin thermal field $\mathbf{H}_{\text{T}}$. This thermal field is an isotropic Gaussian white-noise vector with variance $\sigma^2 = 2\alpha k_{\text{B}}T/(\mu_0 M_{\text{s}} V\tau)$ with k_{B} the Boltzmann constant, T the absolute temperature, V the free layer volume, and τ the simulation time step. This particular stochastic

contribution can be shown to yield appropriate thermal fluctuations[11].

Supplementary Equation (3 is solved numerically using an implicit midpoint rule scheme[12]. The SHE current pulse lasts for 10 ns, after which the simulation is continued for another 10 ns to allow for cooling. The magnetization is considered switched if the final magnetization vector **M** has a **z**-component opposite in sign to the starting condition.

We set $K_{\mathrm{U}} = 4.33 \times 10^5\ \mathrm{Jm^{-3}}$ to yield a thermal stability of $\Delta \equiv K_{\mathrm{eff}}V/(k_{\mathrm{B}}T) = 60$ at room temperature, for a bit size of 100 nm x 100 nm x 0.7 nm. Here, $K_{\mathrm{eff}} = 3.55 \times 10^4\ \mathrm{Jm^{-3}}$ is the effective anisotropy after correcting for the demagnetization field. This value is significantly lower than the experimentally determined value (see Supplementary Note 1) to account for micro-magnetic effects such as domain formation, improving the correspondence between simulations and experiments. Other notable parameters include $\alpha = 0.2$, $M_{\mathrm{s}} = 1.0 \times 10^6\ \mathrm{Am^{-1}}$ for Co7, and $\theta_{\mathrm{SHE}} = 0.07$ for Pt7.

As mentioned in the main text, the local structure of the anti-ferromagnetic material can be approximated in simulations. Two modifications can be implemented, as discussed in the main text and elucidated below. The averaged effect of these local variations is computed by averaging over 256 simulations, each with different local exchange bias parameters.

First, the exchange bias direction can locally vary from the field-cooling direction. To simulate this, we draw the exchange bias direction from a uniform random distribution over the surface of a sphere. We then collapse this distribution between $-\pi/4 < \theta < \pi/4$ and $-\pi/4 < \psi < \pi/4$, where θ and ψ are the azimuthal and elevation angle with respect to the $\hat{\mathbf{y}}$ direction, respectively. The resulting distribution is constrained to 45° offset angles from the $\hat{\mathbf{y}}$ direction, as illustrated in

Supplementary Figure 15. This simulates the field-cooling procedure, where the exchange bias direction is forced to the nearest cubic axis of each grain in the poly-crystalline material.

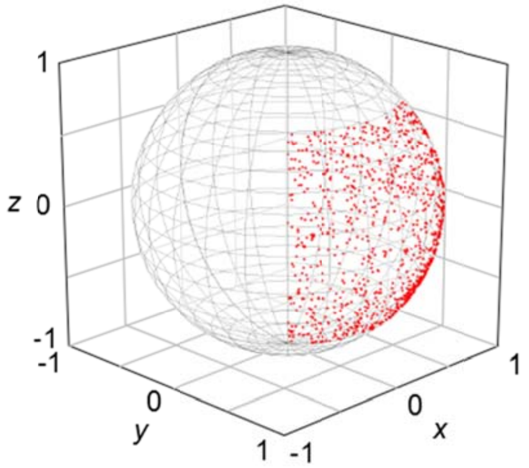

Supplementary Figure 15: Exchange bias direction distribution. The direction of the exchange bias field is chosen from a uniform distribution between certain angles and plotted on the surface of a unit sphere

Second, we implement variations in the local exchange bias magnitude (due to grain size variations, for instance) by drawing it from a chi-distribution with 3 degrees of freedom ($\chi_{3,}$ similar to the Maxwell-Boltzmann distribution) as plotted in Supplementary Figure 16. The width of this distribution is chosen such that the simulated phase diagrams parallel and perpendicular to the exchange bias direction display a vertical shift similar to the one observed in experiments.

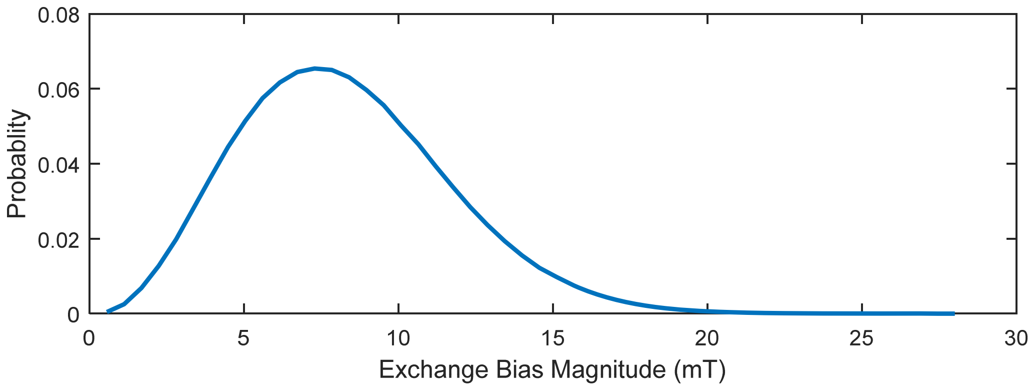

Supplementary Figure 16: exchange bias magnitude distribution. The probability (arbitrary units) of a certain exchange bias magnitude is computed by drawing from a χ-distribution with three degrees of freedom.

Finally, note that our choice of distributions for the exchange bias direction and magnitude should be considered an Ansatz based upon our experimental data. Our model suffices to explain the important trends observed in our experiments (see Supplementary Note 6), but the agreement is not perfect. This is particularly visible in the imperfect reproduction of the shape of the high-probability switching region along the exchange bias direction (c.f. Figure 4a and Figure 4c, main text). Further research may result in a more accurate description of the local exchange bias parameters.

Supplementary References

[1] King, J. P., Chapman, J. N., Gillies, M. F. & Kools, J. C. S. Magnetization reversal of NiFe films exchange-biased by IrMn and FeMn. *J. Phys. D. Appl. Phys.* **34**, 528–538 (2001).

[2] Gogol, P., Chapman, J. N., Gillies, M. F. & Vanhelmont, F. W. M. Domain processes in the magnetization reversal of exchange-biased IrMn/CoFe bilayers. *J. Appl. Phys.* **92**, 1458–1465 (2002).

[3] O'Grady, K., Fernandez-Outon, L. E. & Vallejo-Fernandez, G. A new paradigm for exchange bias in polycrystalline thin films. *J. Magn. Magn. Mater.* **322**, 883–899 (2010).

[4] Xi, H. & White, R. M. Theory of the blocking temperature in polycrystalline exchange biased bilayers based on a thermal fluctuation model. *J. Appl. Phys.* **94**, 5850–5853 (2003).

[5] Chen, Y.-T., Lin, Y. C., Jen, S. U., Tseng, J.-Y. & Yao, Y. D. Effect of Ta seed layer on crystalline structure and magnetic properties in an exchange-biased Co/IrMn system. *J. Alloys Compd.* **509**, 5587–5590 (2011).

[6] Nogués, J. & Schuller, I. K. Exchange bias. *J. Magn. Magn. Mater.* **192**, 203–232 (1999).

[7] Liu, L., Lee, O. J., Gudmundsen, T. J., Ralph, D. C. & Buhrman, R. A. Current-Induced Switching of Perpendicularly Magnetized Magnetic Layers Using Spin Torque from the Spin Hall Effect. *Phys. Rev. Lett.* **109**, 096602 (2012).

[8] Zhang, W. *et al.* Spin Hall Effects in Metallic Antiferromagnets. *Phys. Rev. Lett.* **113**, 196602–196608 (2014).

[9] van den Brink, A. *et al.* Spin-Hall-assisted magnetic random access memory. *Appl. Phys. Lett.* **104**, 012403 (2014).

[10] Koch, R. H., Katine, J. A. & Sun, J. Z. Time-Resolved Reversal of Spin-Transfer Switching in a Nanomagnet. *Phys. Rev. Lett.* **92**, 088302 (2004).

[11] Brown, W. F. J. Thermal fluctuations of a single-domain particle. *Phys. Rev.* **130**, 1677–1686 (1963).

[12] d'Aquino, M., Serpico, C., Coppola, G., Mayergoyz, I. D. & Bertotti, G. Midpoint numerical technique for stochastic Landau-Lifshitz-Gilbert dynamics. *J. Appl. Phys.* **99**, 08B905 (2006).
